# Child Life in London

**Published:** 1895-09-21

**Authors:** 


### Social Problems.

### CHILD LIFE IN LONDON.

This month is the time for estimating the benefit of the "country holiday" to little Londoners. It is easy to pick out of a family the one who has been away, and the difference revealed is often so striking that the question suggests itself what elements in the London life, as contrasted with that in the country, combine so seriously to affect the vitality of the children.

Change of air is, of course, a very large factor in improvement of health; children removed from airless dwellings, and streets only a degree less stifling than the rooms, respond as readily to the renewal of oxygen as a dying lamp drawn up out of the foul air it is testing. But it must be remembered that in some parts of London the death-rate is a good deal lower than that of the places to which the children are sent, and the air is almost beyond reproach; the wide grassy stretches of Regent's Park, of Hampstead Heath, of Victoria Park, and the like, afford healthy playgrounds such as many country children lack, and the happy outdoor life enjoyed in these parks by thousands of youngsters can be noted by any observer. Moreover, sanitation is as a rule much better in London; "country smells" are proverbial, and far more noxious than the fried fish odours of the London slum, and the London tenement house is generally less damp and less draughty than the average labourer's cottage.

Mr. H. Dendy, in the recent volume on social subjects, edited by Mr. Bosanquet,* rightly places much of the deterioration of the London child to the score of the perpetual excitement of the street life. "If you look closely you will see that London children are always tired; the dark rings under their eyes tell of the nervous strain which is breaking down their health; and their very restlessness is the restlessness of fatigue and nervous exhaustion." The alertness of mind, so great a charm in the little street Arab, the readiness of comprehension and repartee, are developed at an enormous expenditure of brain force, which tells on the whole system. The restfulness of country quiet to the little tired brains, taxed to the utmost in school and incessantly diverted to new objects outside, is sufficient of itself to account for the new look of youth on these little returning pilgrims. Think, too, what the long quiet hours of sleep in a pure atmosphere mean to children accustomed to scour the streets nightly till eleven or even twelve o'clock, or who at best scramble earlier to their scanty share of the family bed, in a room pervaded by the cooking, talk, and occupations of all the rest, to be awakened in a few hours by the departure of one or other to early work. It is strictly true of thousands of London children that they never get a really sufficient amount of sleep. I have not spoken of the burden of care resulting from the charge of younger brothers and sisters, or the drunkenness of parents, or the ceaseless small anxieties springing out of precarious employment, because to these the country child is almost equally exposed, but they help largely to increase the evil and to maintain that tension of the nerves which seldom knows complete relaxation.

It is very difficult to compare the town and country ways of feeding. Not only is food much cheaper in London, but wages being higher, more money is expended per head on food. It would seem, therefore, that the London child must be better fed than the country one, and as regards the respectable class of working people, however poor, this may be regarded as true. Among the class now euphemistically known as the "industrial residuum," from which a large proportion of country holiday children are taken, the children are undeniably ill-fed, but it does not follow that this is altogether a question of money. A visitor commenting once on the appearance of a new "sweet" shop in a notoriously poor street, was informed by the owner "that children of really poor people had always money to spend, quite unlike those who had a nasty kind of pride and called themselves respectable, and yet had never a penny to spare for the sweet-shop." The habit of self-indulgence begun in babyhood never dies out. The children spend lavishly on sweets, fruit, nuts, ices, &c., which load the digestion and impair the appetite, while the parents, knowing no better, sacrifice wholesome food to a craving for cheap pastry, pickles, and anything highly seasoned they can procure. The meals are not only fanciful, but highly irregular. The children run about indiscriminately with "pieces" cut whenever they clamour, and the waste is incalculable. Mr. Dendy's description is only too true. "They are generally sucking sweets of some description, and they are nearly always one behind with their meals. It is quite true that many of them come to school without having breakfasted, and this is because their parents interpret too literally the maxim of 'sufficient unto the day.' They empty their cupboard every day, and have to earn a breakfast before they can eat it; and though the children are always late for school the household is seldom sufficiently advanced in its operation to feed the children before turning them out." For the ill-fed, and therefore sickly children of this class, the plain regular meals of the country household are among the most important of the benefits derived from their country holiday.

Plainly, it is the habits far more than the air of the slum household which need regeneration. More sleep, wholesome food, less crowded bed-rooms, and quieter, more self-restrained lives are what the children need if they are to keep the healthy looks they are bringing back with them after a few days only of a more healthful environment. And to this end the social reformer must work, satisfied that country holidays undo much evil which might otherwise become irreparable, create a taste for purer pleasures, and exercise a far-reaching effect for good on the public health of the community.

* "Aspects of the Social Problem by Various Writers." Edited by B. Bosanquet. (Macmillan and Co.)

